# Deterioration of Mechanical Properties and the Damage Constitutive Model of Corroded Steel in an Industrial Environment

**DOI:** 10.3390/ma15248841

**Published:** 2022-12-11

**Authors:** Zongxing Zhang, Yuxuan Xu, Guangchong Qin, Shanhua Xu, Rou Li

**Affiliations:** 1 Jiangsu Key Laboratory of Environmental Impact and Structural Safety in Engineering, China University of Mining & Technology, Xuzhou 221116, China; 2 China Shipbuilding Industry Corporation International Engineering Co., Ltd., Beijing 100021, China; 3 School of Civil Engineering, Xi’an University of Architecture and Technology, Xi’an 710055, China

**Keywords:** corrosion, steel, mechanical properties, damage mechanics, constitutive model

## Abstract

To investigate the degradation law of the mechanical properties of corroded steel, the standard specimens from machining steel members in service for 9 years in an industrial environment were subjected to tensile tests. The influences of different degrees and types of corrosion on the fracture path, stress-strain curve, and mechanical properties of specimens were discussed. Finally, the damage constitutive model of corroded steel was established based on the damage mechanics theory. The results showed that the failure modes of corroded specimens were related to the degrees and types of corrosion. The fracture morphology of specimens with general corrosion were step-like and the fractures of steel were uneven. However, those with local corrosion were mainly flat-like, and the fracture path was along the cross section where the larger corrosion pits were located. The fracture path of the specimen was related to the interaction of the corroded surface and internal material defects (holes). Meanwhile, with the increase of corrosion degree, the yield platform of stress-strain curve gradually became shorter, or even disappeared, and the ultimate strain and elongation at break decreased, implying that the ductility of steel became worse. Ultimately, the good agreement between the curves of the model and test indicated that the damage model could reflect the damage development process of corroded steel in the tensile process better. Corrosion damage resulted in the decrease in the damage threshold, and the damage variable *D* decreased by the time fracture occurred and the maximum reduction rate was up to 62.5%.

## 1. Introduction

With the advantages of high strength, simple fabrication and superior mechanical properties, steel structure, compared with other materials, has become the preferred structural type of civil buildings, industrial buildings and long-span bridges. With the increase of service time, steel structures will suffer various types of damage, and corrosion is one of the representative types of aging damage of steel structures [1,2,3]. Based on the external investigation of cable-stayed bridges, Stafford and Watson [4] found that cable corrosion existed in nearly 100 bridges, which seriously affected the safe operation of bridges. In addition, the global economic loss caused by corrosion of steel structure is nearly 2.2 trillion USD every year [5]. The corrosion of steel structure not only causes huge economic losses, but also leads to engineering accidents and threatens people’s lives and property safety [6,7,8]. For example, severe corrosion of the Kinzua steel bridge in Pennsylvania led to its instantaneous collapse under wind load [9]. Corrosion also caused the entire roof structure of an industrial workshop to collapse in 2020. The accident site was shocking, but the collapsed structure had only been put into use for three years. As a consequence, it is of great practical significance to investigate the mechanical properties of corroded steel structures in service [10,11].

Research on the mechanical properties of corroded steel were the basis of performance evaluations of corroded steel structures in service. At present, plenty of research on the tensile mechanical properties of corroded steel have been carried out. Guedes et al. [12] established the relationship between mechanical indexes, such as elastic modulus, yield strength and ultimate strength of corroded steel, and volume loss rate by the monotone tensile test of corroded steel in a marine environment. Nakai et al. [13,14,15] studied the influence of corrosion on various mechanical properties of steel using artificial drilling to simulate corrosion pits. The results showed that the strength and ductility of corroded steel decreased sharply with the increase of pit depth, and the influence of non-uniform corrosion on the strength of steel was more than that of uniform corrosion.

Paik [16] used section loss to characterize the corrosion degree of steel plates, and determined the quantitative relation between the residual strength of corroded steel plate and the loss rate of cross-sectional area. Wang et al. [17] investigated the influence of corrosion on the mechanical properties of steel, and proposed a numerical method based on surface morphology to predict the residual properties of corroded steel. Appuhamy et al. [18] believed that corroded steel plates tended to fail at the minimum section. Therefore, effective thickness including maximum thickness loss was proposed to predict the residual yield strength and ultimate strength of steel. In a word, previous studies showed that corrosion damage had a great impact on the mechanical properties of steel; however, the corroded steel specimens were mostly processed using artificial machinery, or accelerated by artificial corrosion, and compared with the steel dismantled in actual engineering, there was still huge disparity [19,20,21]. 

In addition, researchers based their functional relationship curves on the mechanical property parameters of corroded steel and an evaluation index of corrosion degree, while studies on the damage constitutive model of corroded steel were relatively few. At present, there is a lack of accurate curves to describe the relationship between stress and deformation of corroded steel and an accurate constitutive model suitable for numerical analysis of corroded steel [22,23,24]. Therefore, it is necessary to systematically investigate the degradation law of mechanical properties and corrosion damage constitutive model of corroded steel.

In this paper, the standard specimens for machining of steel members, in service for 9 years in an industrial environment, were subjected to tensile tests in order to discuss the influences of different degrees and types of corrosion on the fracture path, stress-strain curve and mechanical properties of the specimens. On this basis, combined with the theory of damage mechanics, the damage constitutive model of corroded steel was established, which laid a theoretical foundation for the prediction of mechanical properties and accurate numerical analysis of corroded steel.

## 2. Experimental Methodology

### 2.1. Specimen Design

**Figure 1:**
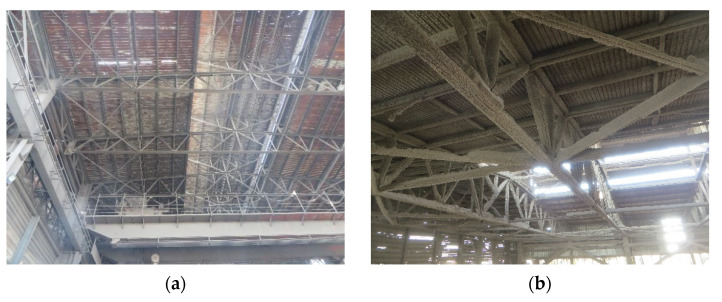
Source of specimen: (**a**) industrial plant and (**b**) working environment.

The specimen was derived from C-shaped steel purlins that had been in service for 9 years in an industrial environment (high temperature (>40 °C) and humidity (>75%) environment). In the production process, due to a great deal of waste steam and dust (SO_4_^−^ and Cl^−^) discharged through the skylight at the top of the main factory building, the aging of the coating of steel components was accelerated and its protective effect was reduced, which led to serious corrosion of steel structure in the long-term service process. It can be seen in Figure 1 that the steel purlin surface was seriously deposited and local buckling of some members occurred due to corrosion-induced weakening of the material, which seriously affected the structure security.

**Figure 2:**
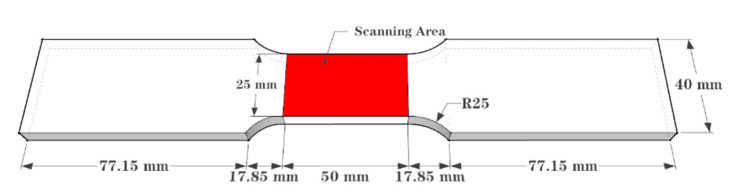
Standard specimen size.

**Figure 3:**
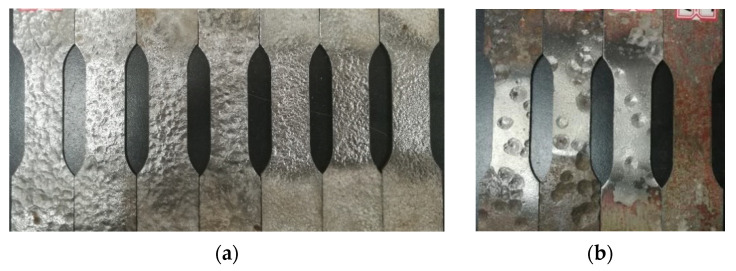
Surface of the specimen after rust removal: (**a**) general corrosion and (**b**) local corrosion.

In order to investigate the degradation law of mechanical properties of steel after corrosion, the paper selected cold-formed thin-wall C-roof steel purlin as the objects of study. Referring to “GB/T 228.1-2010 Tensile test method for metallic materials at room temperature” [25], the standard specimens were cut from the web of steel purlin. Moreover, the corrosion products on the surface of standard specimens needed to be removed by mechanical method, and then immersed in a 12% hydrochloric acid solution for 20 min [26]. Subsequently, the remaining products attached to the surface of specimens were washed by wire ball scrub and ultimately, the surface residue was cleaned with calcium hydroxide solution, washed and dried with clean water, and then cooled to room temperature for weighing. The size of the standard specimen is shown in Figure 2 and the surface state of the specimen after rust removal is shown in Figure 3.

### 2.2. Evaluation of the Degree of Corrosion

**Figure 4:**
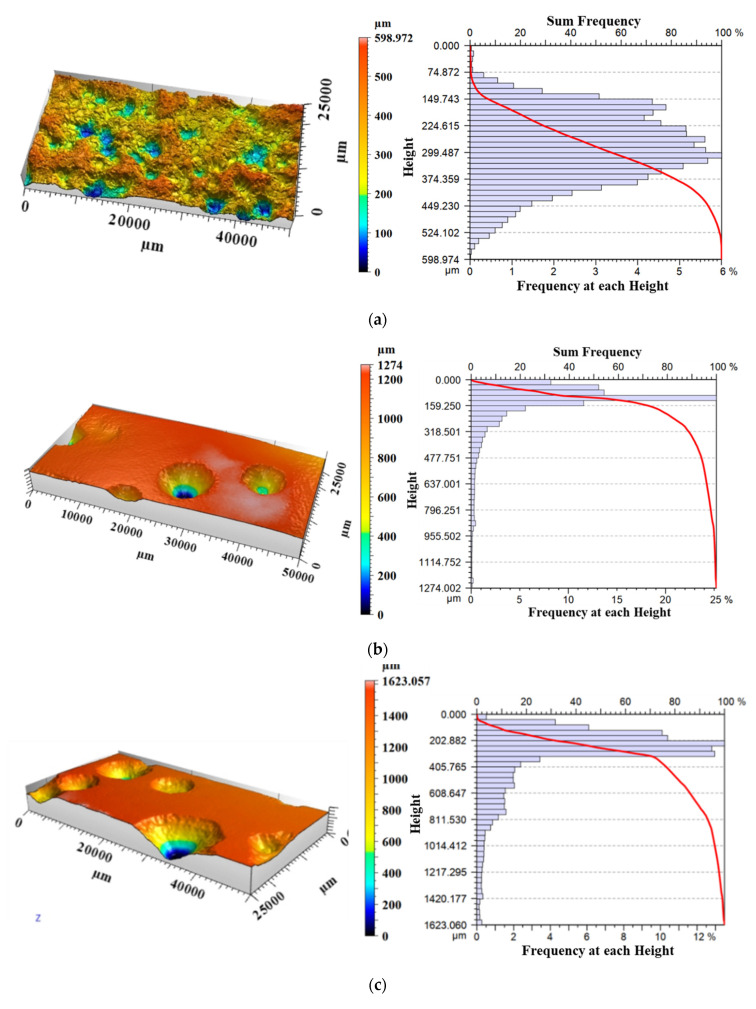

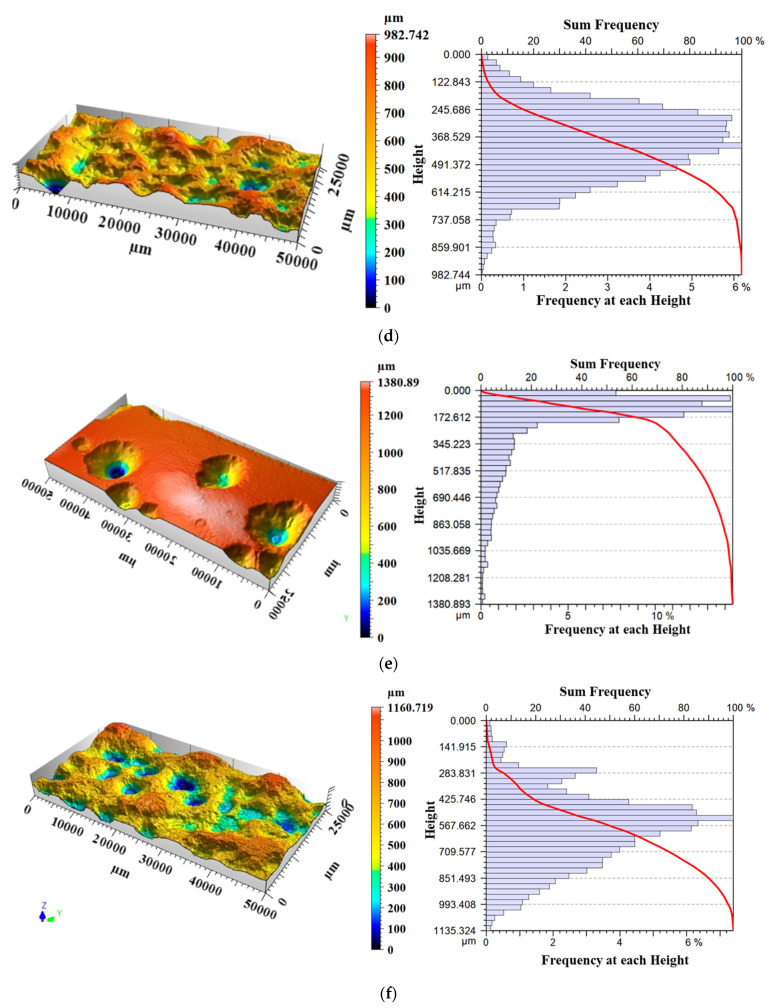
Three-dimensional scanning of the surface of standard specimens: (**a**) A2, (**b**) A6, (**c**) A7, (**d**) A9, (**e**) A10, (**f**) A14.

A three-dimensional non-contact surface topography scanner ST400 produced by American NANOVEA company was used to collect the surface morphologies of standard specimens with different corrosion degrees. The size of the scanning area was 50 mm × 25 mm in the test section of the specimen, as shown in the red area in Figure 2. The scanning results of some representative specimens are shown in Figure 4.

As shown in Figure 4, there were obvious differences in corrosion degree of steel structure surface due to corrosion medium concentration, coating quality and component location. According to the surface morphology, the corrosion types of steel could be divided into general corrosion and local corrosion. The surfaces of specimens were filled with large numbers of semi-ellipsoid etch pits and the size of corrosion pits varied greatly. Moreover, the frequency distribution histogram of local corrosion depth was full in shape and tended to reflect the M-shaped bimodal distribution. Meanwhile, the corrosion degree of specimens A2, A9 and A14 and the depth of pits gradually increased, mainly distributing in the range of 150–400 µm, 200–600 µm and 300–800 µm. For local corrosion, several deep conical pits were scattered on the surfaces of the specimens, and the sizes varied greatly. In addition, the frequency distribution histogram of local corrosion depth was unimodal and its shape was steep. Local corrosion (pitting corrosion) could easily lead to local instability or fracture of components in good overall condition, which was more harmful.

Due to the non-uniformity of local corrosion, the traditional mass loss ratio could not accurately reflect the impact of corrosion pits on the mechanical properties of steel [27,28]. The minimum cross-sectional area *S*_min_ and the average cross-sectional area *S*_s_ were obtained by virtue of three-dimensional scanning data (coordinate points) and MATLAB. Actually, the corrosion degree *D*_s_ of the specimens could be divided into general corrosion index *D*_sa_ and local corrosion index *D*_sn_. The calculation formulas of *D*_sa_ and *D*_sn_ are shown in Equations (1) and (2) and the calculation results of corrosion degree *D*_s_ of the specimens are shown in Table 1.

(1) $$D_{s}{}_{a}=(S_{0}-S_{\mathrm{ave}})/S_{0}$$

(2) $$D_{s}{}_{n}=(S_{0}-S_{\min})/S_{0}$$

where *S*_0_ represents the section area of the non-corroded specimen.

### 2.3. Strength Test

**Figure 5:**
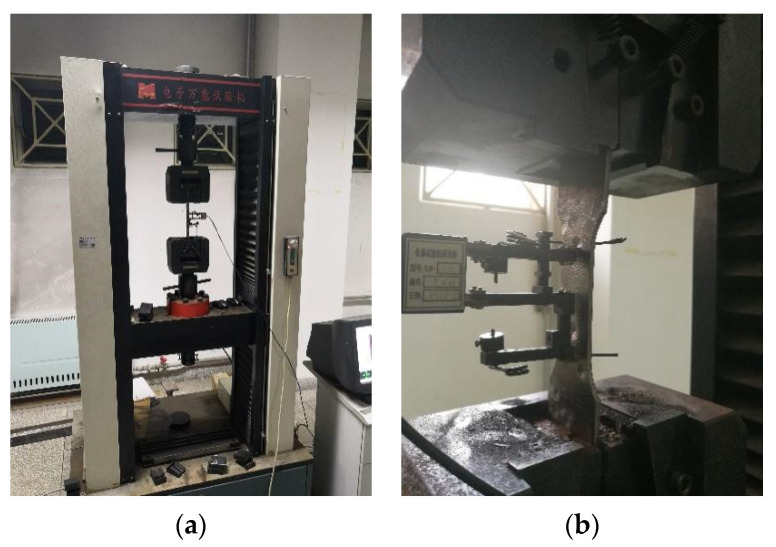
Test loading system: (**a**) the test device and (**b**) test specimen.

The test device was a DNS300 electronic universal testing machine, shown in Figure 5. According to the requirements of the GB/T 228.1-2010 “Tensile test method for metallic materials at room temperature” [25], the loading rate of the elastic stage and yield stage was 0.75 mm/min and that of strengthening stage was set at 5 mm/min. In order to prevent the extensor from being damaged, the loading rate was set at 0.25 mm/min when the load curve dropped and the test would not stop until the specimen broke down.

## 3. Test Results

### 3.1. Failure Modes

**Figure 6:**
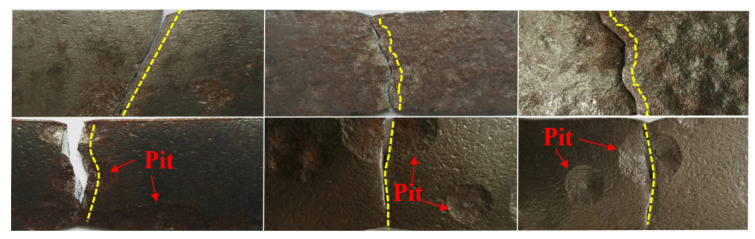
Tensile failure patterns of corroded specimens.

The dissimilitude between the degree and type of corrosion led to diverse failure patterns of the tensile specimen, as shown in Figure 6. The yellow line in the figure is the fracture path of the specimens. It could be found that the non-corroded specimens showed obvious necking and the oblique fracture morphology presented an angle of 45 degrees. When the tensile specimen was corroded, the necking phenomenon gradually disappeared and the fracture path was diversified. In addition, the fracture morphology of the general corrosion specimens was step-like, and with the boost of corrosion, the fracture morphology basically transferred from an oblique fracture to a flat fracture. However, the fracture morphologies of local corroded specimens were mainly flat-like, and the fracture path was along the cross section where the larger corroded pits were located. Actually, the reasons for the diversification of fracture paths were that, with the development of corrosion degree, cross-sectional area loss and surface roughness at different locations were diverse, and the existence of small and unevenly distributed pits gave rise to the phenomenon that the complete net section strength failed to be achieved simultaneously, which caused the specimens to fracture along the weakest section in turn. Ultimately, because of the discrepancy of corrosion morphology, the corroded specimens showed different fractures with various forms.

**Figure 7:**
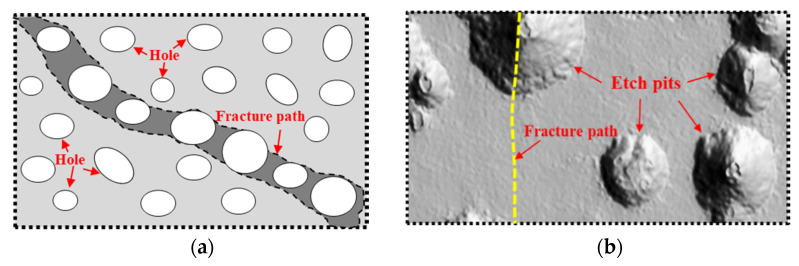
Mechanism of steel fracture expansion: (**a**) matrix holes (ductile fracture) and (**b**) surface pitting (brittle fracture).

Figure 7 shows the mechanism of steel fracture expansion. For non-corroded specimens, dislocation plugging occurred in the inclusions, precipitates, grain boundaries or other plastic deformation discontinuity within the steel matrix, resulting in stress concentration and the formation of micro-void. Furthermore, the plastic strain and average stress made the holes expand, polymerize and even expand gradually, and finally formed a macroscopic crack. Due to the change of elastic-plastic constraints, the maximum shear plane (about 45 degrees from the tensile direction) became the most easily deformable surface, and with the further growth of the holes, the oblique fracture was formed, as shown in Figure 7a.

For corroded specimens, the distribution of corrosion pits on the steel surface made the stress distribution heterogeneous, while the surface stress of general corroded specimens had little difference. Under tensile load, the existence of corrosion pits on the steel surface caused the local plastic strain concentration and stress distribution change, which put an impact on the growth and evolution of micro-void in the area around the pits. The joint action of internal holes in the matrix and distributed pits on the surface led to the change of steel fracture mechanism and the deterioration of fracture toughness. With the increase of corrosion, the fracture form of the specimen changes from oblique fracture to flat fracture. As for the local corroded specimens, the surface stress distribution is extremely uneven, forming obvious weak sections. As a result, cracks, under tensile load, appeared when the section strain reached the ultimate strain due to the large stress around the crater, which meant the section could not bear the load any longer and brittle fracture occurred. Then a flat fracture was formed along the section where the crater was located, as shown in Figure 7b.

### 3.2. Analysis of Mechanical Properties

**Figure 8:**
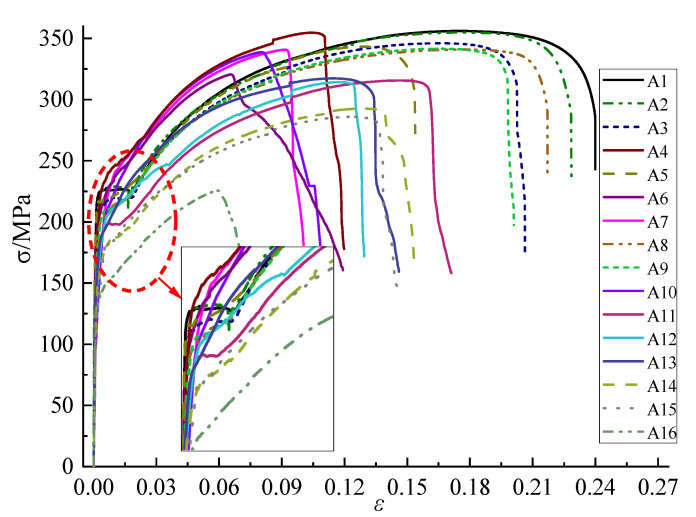
Stress-strain curves of corroded specimens.

Stress-strain curves of specimens with different corrosion types and degrees are shown in Figure 8. It was found that with the increase of corrosion degree, the yield platform of the specimens gradually became shorter or even disappeared. Meanwhile, the ultimate strain and elongation at break decreased and the ductility of the specimens became worse, which was reflected in the local and general corroded specimens with severe corrosion. Actually, it was mainly due to corrosion that the development of stress in the section was not synchronous, and the different positions of the same section showed a gradual yield trend, which was manifested in the disappearance of the yield platform in the stress-strain curve. On the other hand, the severe local corrosion of the specimens resulted in significant local stress concentration, which made the stress fail to be redistributed within the plastic range, and then brittle failure occurred prematurely.

**Figure 9:**
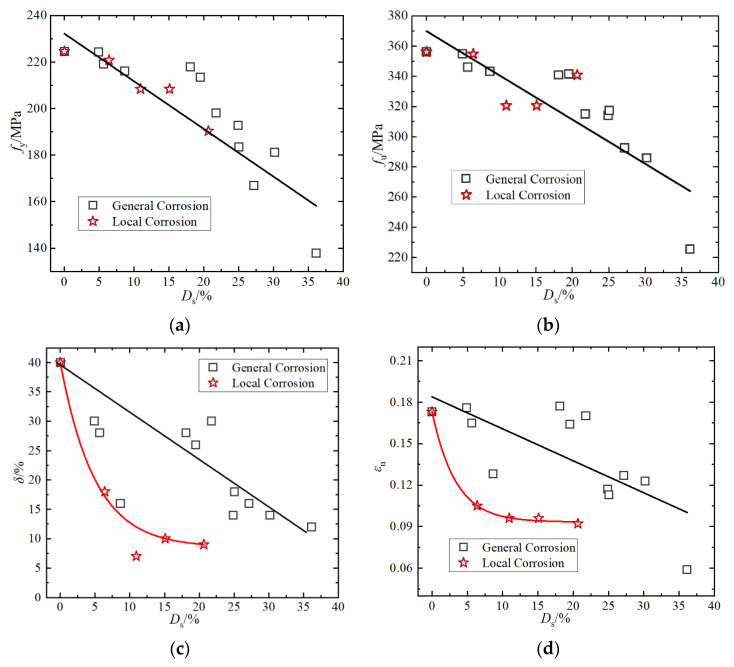
Relationship between mechanical properties of corroded steel and the degree and type of corrosion: (**a**) yield strength, (**b**) ultimate strength, (**c**) elongation, and (**d**) ultimate strain.

Figure 9 shows the relationship between the mechanical properties of different specimens and the degree and type of corrosion. The steel strength index (yield strength and ultimate strength) has nothing to do with the type of corrosion, but is only related to the degree of corrosion; with the increase of the degree of corrosion, the steel strength gradually decreased. The ductility index (elongation and ultimate strain) of steel is related to the type and degree of corrosion. For general corrosion, the degradation law of elongation and ultimate strain was approximately linear with the degree of corrosion. However, for local corrosion, the elongation and ultimate strain of the specimen decreased sharply with the increase of the degree of corrosion. For example, when the corrosion rate is 15.1%, the ultimate strain and the elongation of the specimens decreased by 44.5% and 75%, respectively.

## 4. The Damage Constitutive Model of Corroded Steel

### 4.1. Damage Variables

When the cross-sectional area of steel in the non-destructive state is *A*, and the area after damage is $\tilde{A}$, then the damage variable *D* under the stress state is defined as:

(3) $$D=\frac{A-\tilde{A}}{A}=1-\frac{\tilde{A}}{A}$$

When *D* equals 0, the material is in a non-destructive state. When D equals 1, the material completely breaks. 0 < *D* < 1 indicates the damage state at different degrees. However, due to corrosion damage, the effective section is reduced and according to Equation (3), the effective area is expressed as:

(4) $$\tilde{A}=\left(1-D\right)A$$

Therefore, the effective stress of the damaged material is:

(5) $$\tilde{\sigma }=\frac{F}{\tilde{A}}=\frac{F}{\left(1-D\right)A}=\frac{\sigma }{1-D}$$

In the equation, *σ* represents the nominal stress on the cross section of the specimen, *σ* = *F*/*A.*

According to the strain equivalence principle proposed by Lemaitre, the strain of damaged material (*D* ≠ 0) under effective stress is equivalent to that of the same material without loss (*D* = 0). In the case of non-destructive damage, the constitutive equation of the material is expressed as: $\varepsilon =\frac{\sigma }{E}$, and the constitutive equation after damage is obtained by replacing the nominal stress in the equation with the effective stress:

(6) $$\varepsilon =\frac{\sigma }{E\left(1-D\right)}$$

In the equation, *E* represents the elastic modulus, and the damage of the material changed the non-destructive elastic modulus *E* into the elastic modulus with damage:

(7) $$\tilde{E}=E\left(1-D\right)$$

### 4.2. Damage Evolution Equation

According to the general expression of the damage part of dissipative potential:

(8) $$\varphi_{D}^{*}(Y,\overset{•}{p,}\overset{•}{\kappa },\overset{}{T,}D,\varepsilon^{e})=\frac{1}{2}\frac{{\bar{Y}}^{2}}{S_{0}}\frac{\overset{•}{p}+\overset{•}{\kappa }}{{\left(1-D\right)}^{a_{0}}}$$

In the equation, *Y* represents damage energy dissipation rate; $\overset{•}{p}$ represents accumulative plastic strain rate; $\overset{•}{\kappa }$ represents accumulative micro-plastic strain rate; *D* represents damage variable; *S*_0_ and *a*_0_ represents material correlation coefficients.

According to Equation (8), the following equation is obtained:

(9) $$\overset{•}{D}=\frac{\bar{Y}}{S_{0}}\frac{\overset{•}{p}+\overset{•}{\kappa }}{{\left(1-D\right)}^{a_{0}}}$$

Generally, if the cumulative plastic strain rate $\overset{•}{p}$ exists, the micro-plasticity κ can be ignored, and the Equation (9) can transform into:

(10) $$\overset{•}{D}=\frac{\bar{Y}}{S_{0}}\frac{\overset{•}{p}}{{\left(1-D\right)}^{a_{0}}}$$

According to the damage energy dissipation rate:

(11) $$\bar{Y}=\frac{\sigma_{eq}^{2}[\frac{2}{3}(1+\nu )+3(1-2\nu ){\left(\frac{\sigma_{H}}{\sigma_{eq}}\right)}^{2}]}{2E(1-D{)}^{2}}$$

In the equation,$\sigma_{eq}^{}$ is Von Mises stress; $\sigma_{H}$ is hydrostatic pressure; ν is the Poisson’s ratio.

By substituting the plastic yield condition $\frac{\sigma_{eq}}{1-D}-\sigma_{y}=0$ ($\sigma_{y}$ was yield stress) into Equation (11), the following equation is obtained:

(12) $$\bar{Y}=\frac{\sigma_{y}^{2}[\frac{2}{3}(1+\nu )+3(1-2\nu ){\left(\frac{\sigma_{H}}{\sigma_{eq}}\right)}^{2}]}{2E}$$

Therefore, the damage evolution equation is written as:

(13) $$\overset{•}{D}=\left\{ \begin{array}{l} \frac{\sigma_{y}^{2}[\frac{2}{3}(1+\nu )+3(1-2\nu ){\left(\frac{\sigma_{H}}{\sigma_{eq}}\right)}^{2}]}{2ES_{0}}\frac{\overset{•}{p}}{{\left(1-D\right)}^{a_{0}}}\;\;\;\;\;\;\;\;\;\;\;\;\;\;\;(p\geq p_{0})\\ 0\;\;\;\;\;\;\;\;\;\;\;\;\;\;\;\;\;\;\;\;\;\;\;\;\;\;\;\;\;\;\;\;\;\;\;\;\;\;\;\;\;\;\;\;\;\;\;\;\;\;\;\;\;\;\;\;\;\;\;\;\;\;\;(p<p_{0}) \end{array}\right.$$

Through experimental observation, we can see that the evolution process of damage to strain is linear, so *a*_0_ = 0, and Equation (13) is written as:

(14) $$\overset{•}{D}=\left\{ \begin{array}{l} \frac{\sigma_{y}^{2}[\frac{2}{3}(1+\nu )+3(1-2\nu ){\left(\frac{\sigma_{H}}{\sigma_{eq}}\right)}^{2}]}{2ES_{0}}\overset{•}{p}\;\;\;\;\;\;\;\;\;\;\;\;\;\;\;\;\;\;\;\;\;\;\;\;\;\;(p\geq p_{0})\\ 0\;\;\;\;\;\;\;\;\;\;\;\;\;\;\;\;\;\;\;\;\;\;\;\;\;\;\;\;\;\;\;\;\;\;\;\;\;\;\;\;\;\;\;\;\;\;\;\;\;\;\;\;\;\;\;\;\;\;\;\;\;\;\;(p<p_{0}) \end{array}\right.$$

### 4.3. Equation of Damage Variables

In uniaxial tension, when macroscopic cracks form, then $\overset{}{\frac{2}{3}(1+\nu )+3(1-2\nu ){\left(\frac{\sigma_{H}}{\sigma_{eq}}\right)}^{2}}$ is a constant of 1.0, *D = D_R_*, *p = p_R_*. By integrating Equation (14), we obtain:

(15) $$D=\left\{ \begin{array}{l} \frac{\sigma_{y}^{2}}{2ES_{0}}(p-p_{0})\;\;\;\;\;\;\;\;\;\;\;\;\;\;\;\;\;\;\;\;\;\;\;\;\;\;(p\geq p_{0})\\ 0\;\;\;\;\;\;\;\;\;\;\;\;\;\;\;\;\;\;\;\;\;\;\;\;\;\;\;\;\;\;\;\;\;\;\;\;\;\;\;\;\;\;\;\;\;\;(p<p_{0}) \end{array}\right.$$

Then the corresponding damage value *D_R_* at fracture is:

(16) $$D_{R}=\frac{\sigma_{y}^{2}}{2ES_{0}}(p_{R}-p_{0})$$

By substituting Equation (16) into Equation (15), we obtain:

(17) $$D=\left\{ \begin{array}{l} D_{R}\frac{\left(p-p_{0}\right)}{p_{R}-p_{0}}\;\;\;\;\;\;\;\;\;\;\;\;\;\;\;\;\;\;\;\;\;\;\;\;\;\;(p\geq p_{0})\\ 0\;\;\;\;\;\;\;\;\;\;\;\;\;\;\;\;\;\;\;\;\;\;\;\;\;\;\;\;\;\;\;\;\;\;\;\;\;\;\;\;(p<p_{0}) \end{array}\right.$$

Due to the formula $\frac{p_{0}}{p_{R}}=\frac{\varepsilon_{0}}{\varepsilon_{R}}$ existed under uniaxial stress, the Equation (17) is written as:

(18) $$D=\left\{ \begin{array}{l} D_{R}\frac{\varepsilon -\varepsilon_{0}}{\varepsilon_{R}-\varepsilon_{0}}\;\;\;\;\;\;\;\;\;\;\;\;\;\;\;\;\;\;\;\;\;\;\;\;\;\;(\varepsilon \geq \varepsilon_{0})\\ 0\;\;\;\;\;\;\;\;\;\;\;\;\;\;\;\;\;\;\;\;\;\;\;\;\;\;\;\;\;\;\;\;\;\;\;\;\;\;\;\;(\varepsilon <\varepsilon_{0}) \end{array}\right.$$

The corrosion damage model can be determined by virtue of the steel tensile constitutive model without considering corrosion damage; the damage variable *D* was added on the basis of this model, namely the constitutive relation of corroded steel. According to the characteristics of the stress-strain curve, the curve was divided into three sections. Meanwhile, the damage development was not considered in the proportional stage, but if the material entered the plastic stage, the damage development would be considered. Therefore, the constitutive model of steel with corrosion damage can be stated as follows, where *K* and *m* are material constants.

(19) $$\begin{array}{c} \sigma =\left\{ \begin{array}{l} E\varepsilon \;\;\;\;\;\;\;\;\;\;\;\;\;\;\;\;\;\;\;\;\;\;\;\;\;\;\;\;\;\;\;\;\;\;\;\;\;\;\;\;\;\;\;\;\;\;(\varepsilon <\varepsilon_{y})\\ f_{y}\;\;\;\;\;\;\;\;\;\;\;\;\;\;\;\;\;\;\;\;\;\;\;\;\;\;\;\;\;\;\;\;\;\;\;\;\;\;\;\;\;\;\;\;\;\;\;(\varepsilon_{y}\leq \varepsilon \leq \varepsilon_{0})\\ f_{y}+(1-D)K{\left(\varepsilon -\varepsilon_{0}\right)}^{m}\;\;\;\;\;\;\;\;\;(\varepsilon_{0}\leq \varepsilon \leq \varepsilon_{R})\\ 0\;\;\;\;\;\;\;\;\;\;\;\;\;\;\;\;\;\;\;\;\;\;\;\;\;\;\;\;\;\;\;\;\;\;\;\;\;\;\;\;\;\;\;\;\;\;\;\;\;(\varepsilon >\varepsilon_{R}) \end{array}\right. \end{array}$$

### 4.4. Parameters of Corrosion Damage Model

**Figure 10:**
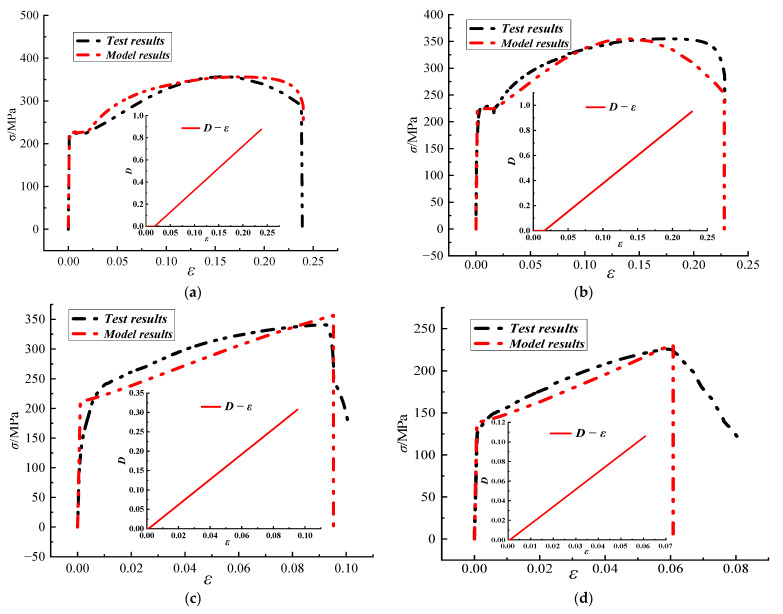
Comparison results between theoretical model curves and experimental stress-strain curves of different corroded specimens: (**a**) A1, (**b**) A2, (**c**) A10, and (**d**) A16.

According to the test data of corroded steel, the parameters of the damage model were obtained, as shown in Table 2. The comparison results of theoretical model curves and experimental stress-strain curves from different corroded specimens, as well as the relationship between damage variables and strain of specimens, were shown in Figure 10. It could be seen that the curve of the corrosion damage model was in good agreement with the test curve, indicating that the damage model could reflect the damage development process of the corroded steel during the tensile process better. When the steel reached yield, the damage variable *D* was basically unchanged. After yielding, the damage began to evolve and showed a linear growth trend. Because of the different degrees of corrosion, steel damage threshold presented discrepancy. Furthermore, compared with the non-corroded specimens, the corroded steel had a lower damage threshold of *ε*_0_, and the damage variable *D* decreased when the section reached the fracture limit.

**Figure 11:**
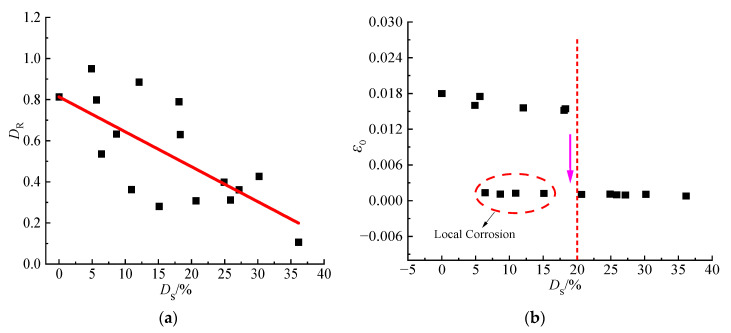
Relationship between corrosion degree, corrosion type and damage threshold ε_0_ and extreme value of damage variable *D_R_*: (**a**) *D*_s_ *− D_R_*. and (**b**) *D*_s_ *− ε*_0._

Figure 11 shows the relationship between the degree of corrosion, the type of corrosion, the damage development threshold and the damage extremum. As seen in Figure 11a, with the increase of the degree of corrosion, the corresponding extreme value of the damage variable showed a downward trend on the whole, and the maximum decrease was as high as 62.5%. Therefore, it was rather unreasonable and extremely dangerous to use the damage development of non-corroded steel to characterize that of steel after corrosion damage, which was to evaluate the degradation of steel mechanical properties. Meanwhile, it can be seen in Figure 11b that for the general corrosion, when the degree of corrosion was lower, the damage threshold would present a linear downward trend and the damage threshold decreased sharply and remained stable by the time the degree of corrosion reached 20%. Actually, for local corrosion, the damage threshold was still decreased seriously, even if the degree of corrosion was lower.

## 5. Conclusions

In this paper, the tensile test was conducted to discuss the influence of diverse degrees and types of corrosion on the mechanical properties of steel. Furthermore, the relationship between the fracture path, stress-strain curve, mechanical properties and the corrosion parameters was studied. On this basis, combined with the theory of damage mechanics, the damage constitutive model of corroded steel was established, and the main conclusions were as follows:

(1) The failure mode of corroded specimens was related to the degree and type of corrosion. The fracture of the general corroded specimens was step-like and the cross-section was uneven, while that of the local corroded specimens was mainly flat-like, and the fracture path was along the cross section where the larger corroded pits were located. Corrosion pits caused local plastic strain concentration and stress distribution changes, which affected the growth and evolution of micro-void in the surrounding areas of the corrosion pits. The joint action of pores in the matrix and surface distribution pits led to the change of steel fracture mechanism and the diversification of fracture paths.
(2) With the increase of corrosion degree, the yield platform of stress-strain curve of the specimen gradually became shorter or even disappeared. Meanwhile, the ultimate strain and elongation at break decreased, which meant the ductility of the specimen became worse. For example, when the corrosion rate was 15.1%, the ultimate strain and the elongation of the specimens decreased by 44.5% and 75% respectively. On the one hand, it was mainly due to corrosion that the development of stress in the section was not synchronous, and the different positions of the same section showed a gradual yield trend, which was manifested in the disappearance of the yield platform. On the other hand, the severe local corrosion of the specimens resulted in significant local stress concentration, which made the stress failed to be redistributed within the plastic range, and then brittle failure occurred prematurely.
(3) With the increase of the degree of corrosion, the corresponding extreme value of damage variable showed a downward trend on the whole, and the maximum decrease was as high as 62.5%. There was good agreement between the corrosion damage model curve and the test curve, indicating that the damage model could reflect the damage development process of the corroded steel in the tensile process better. Moreover, the corrosion damage resulted in the decrease of the steel damage threshold *ε*_0_, and the damage variable *D* decreased when the section reached the fracture limit.

## Figures and Tables

**Table 1:** Average section loss rate and weak section area.

Number	*S*_0_/mm^2^	*S*_s_/mm^2^	*S*_min_/mm^2^	*D*_s_/%
A1	75	75	75	0
A2	75	71.33	61.55	4.89
A3	75	70.77	68.79	5.64
A4	75	70.20	70.20	6.40
A5	75	68.51	67.10	8.65
A6	75	70.32	66.80	10.93
A7	75	71.99	63.68	15.10
A8	75	61.43	57.32	18.09
A9	75	60.37	54.54	19.51
A10	75	70.55	59.51	20.66
A11	75	58.67	52.12	21.78
A12	75	56.33	59.99	24.89
A13	75	56.22	51.62	25.04
A14	75	54.63	56.71	27.16
A15	75	52.37	54.78	30.18
A16	75	47.90	57.52	36.14

**Table 2:** Damage model parameters of corroded steel.

Specimen Number	*D*_s_*/*%	*ε_y_*	*ε*_0_	*ε_R_*	*D_R_*	*K*	*m*	*E*/GPa
A1	0	0.00102	0.018	0.238	0.81327	3500	1.25	220
A2	4.89	0.00124	0.01602	0.22817	0.95076	4000	1.25	181
A3	5.64	0.00122	0.01752	0.20200	0.79833	3500	1.25	179
A4	6.40	0.00134	0.00134	0.11014	0.53550	4000	1.25	170
A5	8.65	0.00110	0.01100	0.18366	0.63264	3500	1.25	178
A6	10.93	0.00126	0.00126	0.08284	0.36264	4000	1.25	173
A7	15.10	0.00122	0.00122	0.06972	0.28054	4000	1.25	171
A8	18.09	0.00125	0.01560	0.21681	0.88557	3500	1.25	174
A9	19.51	0.00126	0.01518	0.19782	0.78971	3500	1.25	170
A10	20.66	0.00106	0.00106	0.09528	0.30755	4000	1.25	200
A11	21.78	0.00113	0.01544	0.18838	0.63057	3500	1.25	178
A12	24.89	0.00099	0.00099	0.1190	0.31162	4000	1.25	167
A13	25.04	0.00110	0.00110	0.13346	0.39824	4000	1.25	167
A14	27.16	0.00096	0.00096	0.14087	0.36139	4000	1.25	174
A15	30.18	0.00108	0.00108	0.13654	0.42643	4000	1.25	168
A16	36.14	0.00078	0.00078	0.06080	0.10592	3500	1.25	178
